# Lobular Breast Carcinoma Presenting as an Endometrial Polyp Metastasis: A Report of a Novel Asynchronous Presentation

**DOI:** 10.7759/cureus.96081

**Published:** 2025-11-04

**Authors:** Amy L Rousselot, Zain Aryanpour, Nicole Christian, Scott Kono, Miriam D Post

**Affiliations:** 1 Pathology, University of Colorado - Anschutz Medical Campus, Aurora, USA; 2 Surgery, University of Colorado - Anschutz Medical Campus, Aurora, USA; 3 Breast Surgery, University of Colorado - Anschutz Medical Campus, Aurora, USA; 4 Oncology, University of Colorado - Anschutz Medical Campus, Aurora, USA

**Keywords:** breast cancer, endometrial polyp, gynecology, lobular carcinoma, metastatic breast cancer, pathology, radiology

## Abstract

Uterine metastases from breast carcinoma are rare. We describe an unusual case of occult metastatic lobular carcinoma incidentally identified within an endometrial polyp in a woman with post-menopausal bleeding. She had no prior history of breast malignancy with no primary mass identified during initial workup. Despite thorough investigation, no primary tumor was detected until two years later. This case highlights an atypical asynchronous presentation of metastatic breast carcinoma and emphasizes the importance of maintaining awareness as well as maintaining vigilant long-term clinical follow up in cases of abnormal metastatic patterns.

## Introduction

Breast carcinoma is the most frequently diagnosed malignancy among women and remains a leading cause of cancer-related mortality worldwide. The most common sites of distant metastasis include the bone, lungs, liver, and brain, whereas involvement of gynecologic organs is distinctly uncommon. When metastasis to the female genital tract does occur, it most often affects the ovaries and is more frequently derived from invasive lobular carcinoma (ILC) than from invasive ductal carcinoma (IDC) [1-3].

ILC is characterized by its unique histopathologic and molecular features, including discohesive tumor cells and loss of E-cadherin expression, which contribute to its diffuse infiltrative growth pattern and propensity for metastasis to unusual sites such as the gastrointestinal tract, peritoneum, and gynecologic organs. Despite this predilection, metastatic involvement of the uterus remains rare, and when present, it is typically observed in patients with a known history of breast cancer. These distinct molecular and morphologic features may account for the tumor’s ability to metastasize to unusual locations, making recognition of such patterns essential for accurate diagnosis [1].

We describe an unusual case of a postmenopausal woman with no known history of breast malignancy who underwent endometrial curettage for abnormal uterine bleeding. Histopathologic examination revealed metastatic ILC involving an endometrial polyp, representing an exceptionally rare presentation in which metastatic disease preceded the identification of the primary breast tumor by two years.

## Case presentation

A 66-year-old woman presented with isolated abnormal uterine bleeding (AUB). She had a personal history of multiple myeloma and a family history of breast cancer in her sister, diagnosed at age 48. Otherwise, she had no known personal history of breast malignancy and denied breast pain, nipple discharge, or palpable masses.

**Figure 1:**
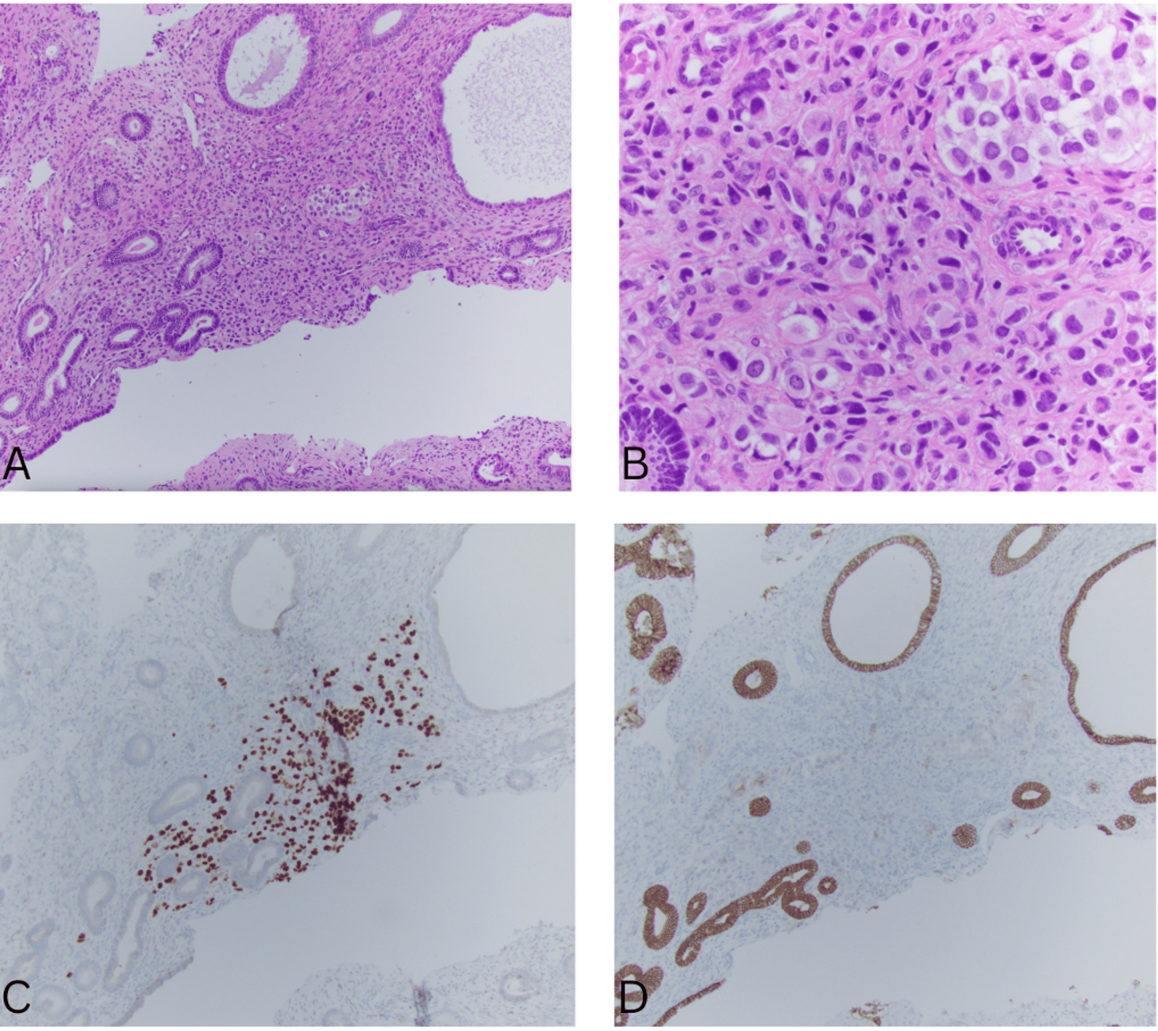
Endometrial polyp. A: H&E, 100x. B: H&E, 400x. C: GATA3 nuclear expression, 100x D: E-cadherin loss, 100x

A thorough gynecologic evaluation, including pelvic imaging, demonstrated findings suspicious for an endometrial polyp. The patient subsequently underwent hysteroscopy, dilation and curettage, and polypectomy. Histologically, the polyp showed an incidental focus (<2 mm) of discohesive atypical cells. Immunohistochemical studies supported a metastatic origin from the breast, showing estrogen receptor and progesterone receptor positivity, with loss of E-cadherin expression (Figure 1).

**Figure 2:**
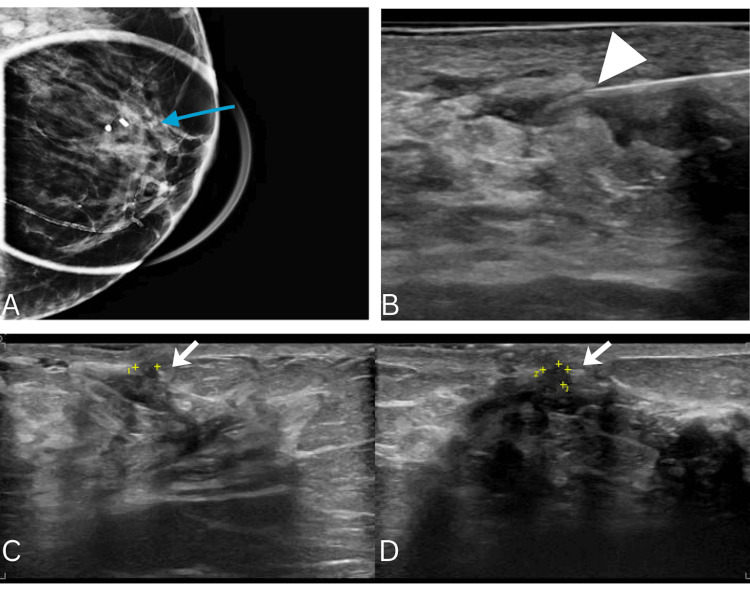
Diagnostic imaging of the left breast. (A) Left breast mammogram showing a suspicious palpable mass (blue arrow). (B) Ultrasound-guided biopsy of the mass (white arrowhead). (C-D) Ultrasound of superficial hypoechoic mass with ill-defined margins, 0.3 x 0.3 x 0.3 cm (white arrows).

**Figure 3:**
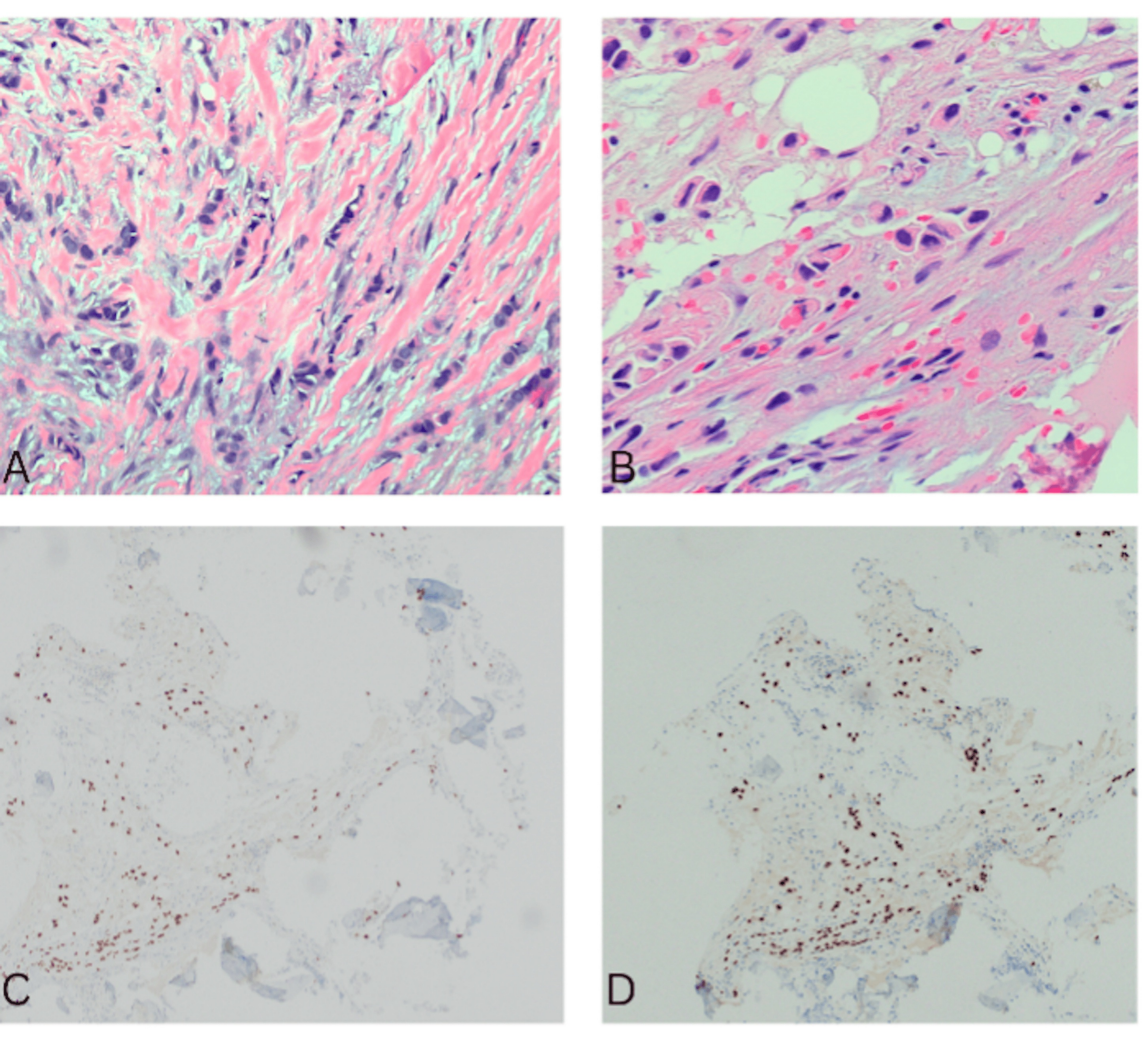
Biopsy of breast and osseous lesions. (A) Ultrasound guided biopsy of breast lesion (H&E, 200x) (B) Osseous lesion (H&E, 400x). (C) Osseous lesion (GATA3, 100x) (D) Osseous lesion (ER, 100x)

**Figure 4:**
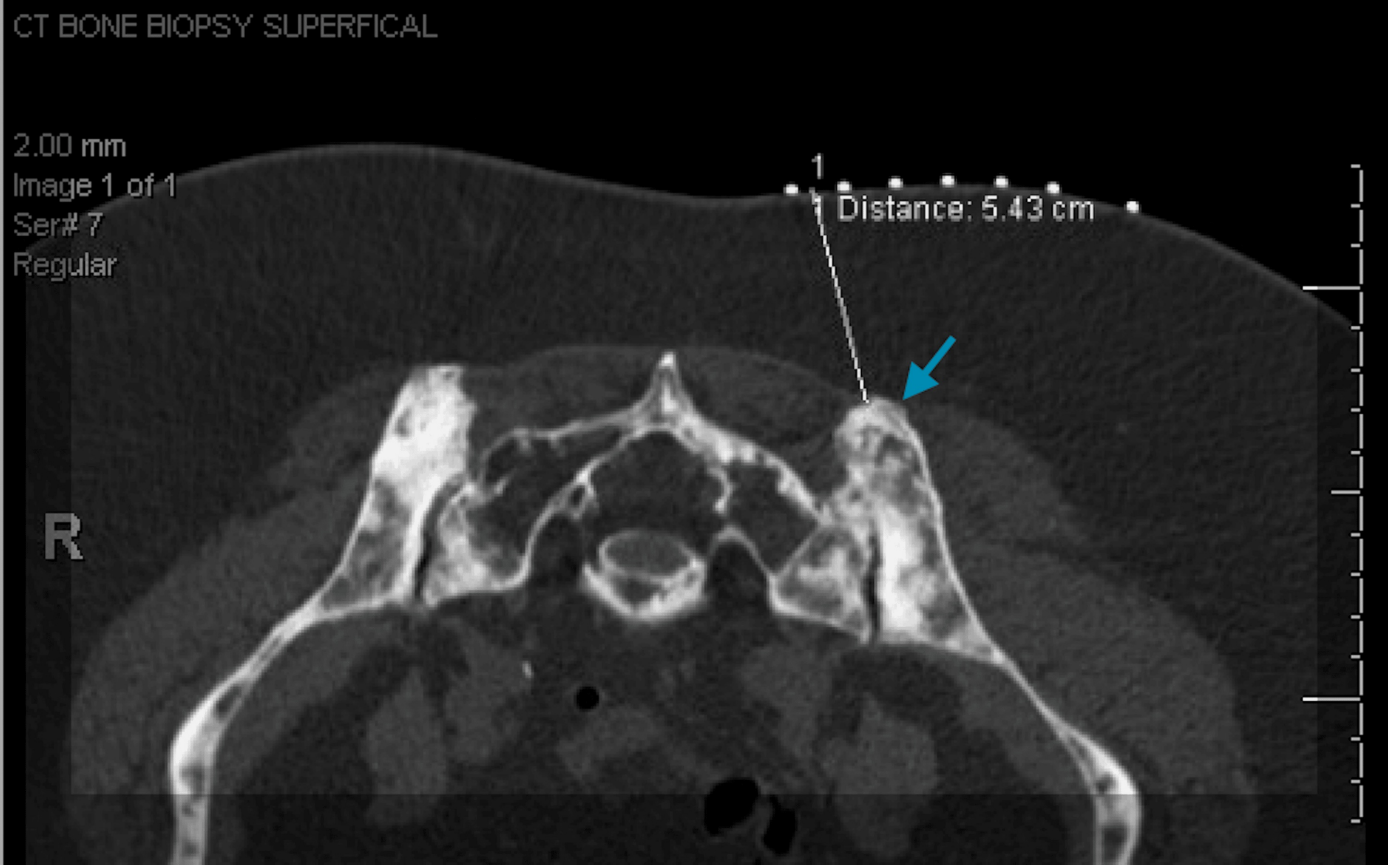
Osseous lesions identified on CT scan. Imaging from the CT-guided targeted biopsy of lytic lesions in the left posterior ischial spine (blue arrow).

Radiographic review performed at the time identified no mammographic or sonographic evidence of malignancy. Given the absence of a detectable primary tumor, the patient was closely followed with interval surveillance imaging. Surveillance subsequently yielded negative results until two years later, when the patient developed a palpable breast mass. Repeat imaging finally revealed a 3 mm lesion (Figure 2A-2D). Core needle biopsy demonstrated ER+/PR+/HER2- ILC (Figure 3A), confirming the primary breast tumor. Follow-up MRI revealed indeterminate bilateral lesions, also confirmed to be ILC. Two months later, PET/CT identified osseous lesions (Figure 4), which on biopsy were positive for metastatic ILC (Figure 3B-3D).

This case is remarkable for the absence of any clinical or radiologic findings suggestive of breast carcinoma at the time of the initial endometrial diagnosis. The metastatic focus was both microscopic and incidentally discovered, underscoring the insidious nature of lobular carcinoma and its potential to metastasize in an atypical, asynchronous pattern. Now, almost five years after the original diagnosis, the patient remains on maintenance therapy with no active disease as of September 2025. 

## Discussion

In the literature, the majority of documented cases of breast cancer metastatic to the uterus occurred in patients with previously diagnosed breast cancer [1]. Though it is rare for breast cancer to present as a metastasis, when it does occur, it is most often lobular carcinoma. In fact, up to 20-40% of patients with invasive lobular carcinoma have metastasis at the time of diagnosis [2]. There is a small cohort of published cases with similar presentations, but all these reports involve patients presenting with either breast cancer symptoms or prior breast pathology [3-10]. There exist only three previously reported cases of patients with no known breast pathology who presented with AUB and were found to have metastatic breast cancer in the uterus [4, 10-12]. In those cases, however, the primary breast tumor was immediately discovered following the diagnosis of metastatic disease (Table 1).

**Table 1:** Literature review of breast cancer presenting as uterine metastases. AUB: abnormal uterine bleeding

Age (years)	Presentation	Detection of primary breast cancer	Histology	Biomarker Status	Distant Metastases	Reference Number
66	AUB	Two years later	Invasive lobular carcinoma	ER+, PR+, HER2-	Endometrium, bone	(this case)
62	Abnormal mammogram, abnormal pap test	Synchronous	Invasive lobular carcinoma	ER+, PR+, HER2-	Endometrium, uterine cervix	[2]
42	Breast mass and uterine enlargement	Synchronous	Invasive ductal carcinoma	ER+, PR+, HER2+	Endometrium, ovary	[2]
53	Breast mass and AUB	Synchronous	Invasive lobular carcinoma	Not reported	Bone, uterine cervix	[7]
49	AUB, endometrial polyp, fibroid uterus	Weeks later	Invasive lobular carcinoma	ER+, PR+, HER2-	Endometrium, myometrium, uterine cervix, ovaries	[8]
47	AUB, fibroids, cholecystitis	Synchronous	Invasive lobular carcinoma	ER+, PR+, HER2-	Endometrium, fibroids, bone, gallbladder, peritoneum	[3]
44	AUB	Weeks later	Invasive lobular carcinoma	Not reported	Endometrium, uterine cervix, bone	[10]
58	AUB	Synchronous	Invasive micropapillary carcinoma	Not reported	Endometrium	[9]
42	AUB	Synchronous	Invasive ductal carcinoma	ER+, PR+, HER2+	Endometrium, bone, axillary lymph nodes	[11]

To our knowledge, this is the only case in which there was an occult breast cancer and a multi-year delay between the initial diagnosis of invasive lobular carcinoma and the detection of the primary breast cancer, despite frequent screenings. While this patient had no overt "red flag" symptoms suggestive of metastatic disease, her presentation emphasizes the importance of maintaining a broad differential diagnosis when evaluating patients with postmenopausal bleeding. 

This case highlights how effective communication between pathology, oncology, and radiology teams can ensure vigilant clinical surveillance and appropriate follow-up, even when the initial findings are unexpected or seemingly isolated. Further studies exploring the molecular mechanisms that drive the unusual metastatic tropism of ILC and the diagnostic limitations of current imaging modalities may help improve early detection and management strategies for such rare presentations.

## Conclusions

This case represents an exceptionally rare presentation of invasive lobular carcinoma, in which a metastatic focus within an endometrial polyp was identified two years before the primary breast tumor. The asynchronous nature of this presentation broadens the recognized spectrum of metastatic patterns associated with lobular carcinoma and underscores that metastases can occasionally precede detection of the primary lesion. In conclusion, although such occurrences are rare, this case highlights the importance of maintaining a broad differential diagnosis, exercising careful histopathologic evaluation, and having good communication within the clinical team when assessing endometrial specimens from patients presenting with abnormal uterine bleeding. Vigilance in these situations can facilitate timely recognition of metastatic disease and inform appropriate clinical follow-up.
